# Intention to quit and the role of dark personality and perceived organizational support: A moderation and mediation model

**DOI:** 10.1371/journal.pone.0195155

**Published:** 2018-03-29

**Authors:** Luke Treglown, Katarina Zivkov, Anthony Zarola, Adrian Furnham

**Affiliations:** 1 Research Department of Clinical, Educational and Health Psychology, University College London, London, United Kingdom; 2 Zeal Solutions, Nottingham, United Kingdom; 3 Norwegian Business School (BI), Olso, Norway; Chiba Daigaku, JAPAN

## Abstract

This study investigated the role of individual differences (dark personality) and situational factors (perceived organisational support) in explaining intention to quit. Four hundred and fifty-one (50 of which females) ambulance personnel completed three questionnaires (Hogan Development Survey; Perceived Organisational Support Survey; and a single item Intention to Quit measure) as a part of a selection and development assessment. Employees high on Excitable, Sceptical, and Mischievous, but low on Colourful were found to have greater intentions to quit. Additionally, employees high on Excitable, Sceptical, Reserved, and Leisurely, but low on Dutiful and Diligent had lower perceptions of organisational support. Structural Equation Modelling revealed that perceived organisational support plays both a mediating and moderating role on dark personality and intention to quit. Theoretical implications of personality’s role in perceived organisational support and intention to quit are discussed.

## Introduction

Employee turnover is a serious and pressing concern that most, if not all, organizations seem to face at one point or another. Voluntary turnover can mean capable and competent employees quit the organization to work someplace else [1]. This has serious implications for organizational success and has been found to be associated with decreased productivity [2], profitability [3], future revenue growth [4] and decreased customer satisfaction [5].

Researchers and human resource professionals have estimated that the turnover of just one person can cost an organization between 93–200% of that person’s salary—given that employee’s amount of responsibility and skill [6; 7]. Further, turnover can result in a loss of important job-specific knowledge and expertise as well as a possible decrease in morale due to the high levels of frustration that result from being unable to meet job demands [8].

The desire is to stop working for an organization is what literature refers to as turnover intention or intention to quit (ITQ). Intentions are key to determinant of actual behaviour as they can predict a person’s perception and resulting judgment made as a result of those perceptions. McCarthy, Tyrrell and Lehane [9] have argued that intention to quit is the final part in the decision making process.

Understanding the antecedents of turnover intentions is of clear importance to both organisations and research within the organisational psychology [10]. Quitting intentions can be influenced by both environmental factors as well as individual differences [10; 11; 12]. A key situational factor for understanding turnover intentions is the concept of perceived organisational support (POS; e.g. [13]). POS has been defined as “the general belief that an employee’s work organization values their contributions and cares about their well-being” [14, p. 698]. A number of studies have looked at the effects of POS on work outcomes and has found that high POS is associated with things like increased commitment to the organization [15], performance [16], and job satisfaction [17].

POS elicits feelings of gratitude, obligation and trust to the organization that should decrease employee intent to quit. Meta-analyses have reliably found that there is negative relationship between POS and turnover intentions [14; 18]. Specifically, if employees perceive high levels of organizational support, they are far less likely to want to leave that organization. In fact, a meta-analysis by Riggle et al. [18] found that 25% of the variance in turnover intention was accounted for by POS.

Additionally, some studies have suggested that personality has direct effects on turnover intentions while others have suggested there are important mediator and moderator variables in this relationship [12; 19; 20]. Recent work in organisational psychology has emphasised the distinction between ‘bright’ and ‘dark’ taxonomies of personality traits.

‘Bright’ traits are considered to represent how we behave interpersonally when being purposeful, positive, and at our best [21, 22]. The Big Five traits that studies have consistently found to be directly indicative of turnover intention are Conscientiousness and Neuroticism. Conscientiousness has been shown to have a negative relationship with intent to leave [1; 12; 23]. Neuroticism on the other hand seems to have a significantly positive relationship with employee quitting intentions [1; 11; 23]. The other three Big Five factors have not had such consistent result. For extraversion some studies found that it had an inverse effect on turnover intention [12; 23; 24], a positive effect on turnover intentions [25] or no effect at all [1]. Agreeableness also showed similar patterns with some studies finding it to positively predict turnover intentions [25] while Jeswani and Dave [24] found a negative relationship. Other studies have also found no relationship at all [1; 11]. Finally Openness to Experience has been found to have a negative impact on turnover in some instances [11; 12], positive effect on turnover in others [25] or no impact at all [1; 24].

‘Dark’ side traits, however, are those that come out with greater frequency when we are not on our guard and have the ability to hinder our professional and personal lives [26]. These tend to emerge when we are tired, stressed or overworked which deplete our cognitive resources that work to inhibit any maladaptive impulses. Researchers have outlined taxonomies of dark personality in attempt to characterise and define how individuals exhibit interpersonally maladaptive behaviours.

One taxonomy of dark personality is the Hogan Development Survey [HDS; 27], which measures 11 dark side personality traits. These are representations of the maladaptive behaviours that are based on the 11 DMS AXIS-II Personality Disorders (as can be seen in Table 1). Conceptually, the 11 scales fit the Horney [28] three-tiered taxonomy of self-defeating behaviours. The first, *Moving Away from Others*, defines traits where people who are threated by stress and tend to isolate themselves from others when they experience it [28]. People with a penchant to move away from people are more likely to quit their jobs as a result of their wish to escape from their problem [29]. *Moving Against Others* defines traits where people who are confident and want to manipulate and assert their dominance in order to deal with their insecurities [28]. People with a propensity to move against others are more likely to have issues and get into trouble within the organization that may lead to both voluntary and involuntary turnover [30]. Finally, *Moving Towards Others* encompasses people who seek integration and prefer to give up responsibility and be reliant on others to make decision for them [28]. These people seek security by meeting other people’s expectations which is likely to result in them staying within the same organization [14].

**Table 1:** Comparison in Themes between DSM-IV-R Axis-II Personality Disorders and HDS ‘Dark Side’ traits.

Axis-II Personality Disorder	Symptom Themes	HDS ‘Dark Side’ Trait	Trait Themes	Horney (1950) Classification
Borderline	Inappropriate anger; unstable and intense relationships alternating between idealization and devaluation.	Excitable	Moody and hard to please; intense, but short-lived enthusiasm for people, projects or things	‘Moving Away’
Paranoid	Distrustful and suspicious of others; motives are interpreted as malevolent.	Sceptical	Cynical, distrustful, and doubting other’s true intentions	‘Moving Away’
Avoidant	Social inhibition; feelings of inadequacy and hypersensitivity to criticism or rejection	Cautious	Reluctant to take risks for fear of being rejected or negatively evaluated	‘Moving Away’
Schizoid	Emotional coldness and detachment from social relationships; indifferent to praise and criticism	Reserved	Aloof, detached, and uncommunicative; lacking interest in or awareness of the feelings of others	‘Moving Away’
Passive-Aggressive	Passive resistance to adequate social and occupational performance; irritated when asked to do something he/she does not want to	Leisurely	Independent; ignoring people’s requests and becoming irritated or argumentative if they persist	‘Moving Away’
Narcissistic	Arrogant and haughty behaviours or attitudes; grandiose sense of self-importance and entitlement	Bold	Unusually self-confident; feelings of grandiosity and entitlement; overvaluation of one’s capabilities	‘Moving Against’
Anti-Social	Disregard for the truth; impulsivity and failure to plan ahead; failure to conform with social norms	Mischievous	Enjoying risk taking and testing limits; needing excitement; manipulative, deceitful, cunning and exploitative	‘Moving Against’
Histrionic	Excessive emotionality and attention seeking; self-dramatizing, theatrical, and exaggerated emotional expression	Colourful	Expressive, animated, and dramatic; wanting to be noticed and needing to be the centre of attention	‘Moving Against’
Schizotypal	Odd beliefs or magical thinking; behaviour or speech that is odd, eccentric, or peculiar	Imaginative	Acting and thinking in creative and sometimes odd or unusual ways	‘Moving Against’
Obsessive-Compulsive	Preoccupations with orderliness, rules, perfectionism, and control; over conscientious and inflexible	Diligent	Meticulous, precise, and perfectionistic; inflexible about rules and procedures; critical of others’ performance	‘Moving Towards’
Dependent	Difficulty making everyday decisions without excessive advise and reassurance; difficulty expressing disagreement out of fear of loss of support or approval	Dutiful	Eager to please and reliant on others for support and guidance; reluctant to take independent action or go against popular opinion	‘Moving Towards’

Very little research has examined the role of dark personality in predicting turnover intentions. As turnover intentions occur when employees experience greater levels of stress [31], it is possible that the interpersonal behaviours an employee utilises when under stress influence the manifestation of turnover intentions. The current study seeks to further understand the relationship between dark personality and ITQ, both directly and in relation to POS.

Additionally, there has been little research examining the dispositional tendencies to experience POS [14]. In their review, Rhoades and Eisenberg [14] posited that personality traits that promote withdrawal and aggression could inhibit the development of favourable working relationships, reducing POS. A recent study demonstrated that dark personality traits influence employees’ positive workplace attitudes [32], arguing that these traits can serve to undermine the development and maintenance of positive perceptions towards the organisation.

Furthermore, little is understood about how the interaction of personality (particularly dark traits) and POS influence the development of turnover intentions. POS has the possibility to both mediate and moderate the effect of dark personality on ITQ. There is evidence that POS mediates the relationship between turnover intentions and organizational justice [33], the relationship between turnover intentions and abusive supervision [34] as well as the relationship between mentoring and employee turnover intentions [35]. If certain dark personality traits undermine an employee’s POS, this could go on to increase the likelihood of employees developing ITQ (mediation). Alternatively and additionally, the effect of dark personality on turnover intentions could be contingent on certain levels of POS (moderation). Analyses of a structural model that depict these two relationships will offer a theoretical understanding of personality and POS’s role in turnover intentions that has not been noted in the literature before.

## Materials & method

### Participants

The participants of this study included 451 ambulance personnel, of which 401 were males and 50 were female. The mean age of this sample was 39.4 years (SD = 8.33). The data was gathered as a part of a selection and development consultation within the specific organization to identify individuals who may be required to respond to high threat or terror incidences. University College London ethics committee approved the protocol prior to the study. Written and informed consent was provided by all participants was given before engaging in the study, and all participants received feedback on their scores.

### Materials

#### Hogan development survey

The HDS is a 154 item measuring how survey responders interact with the people around them—family, friends and co-workers. Participants are asked to “agree” or “disagree” with the items. The HDS has an average coefficient alpha (Cronbach’s alpha) of .64 (ranging from .50 to .70), with an average test-retest reliability of .68, ranging from .58 (Leisurely) to .87 (Excitable; [36; 37]). However, due to the unavailability of item level data, alphas were not able to be reported for this study.

#### Perceived organizational support survey

POS was measured by the Survey of Perceived Organizational Support (SPOS; [16]). The scale features 9 items each scores on a likert scale from 1 (strongly disagree) to 7 (strongly agree). In this study a final POS score was calculated by adding all the scores of the 9 items. Higher values were an indication of higher levels of POS. The scale is composed of questions about the organization’s valuation of the employee and well-being of employees. The SPOS had a Cronbach’s alpha coefficient of .94. An example item is *‘The [organisation] takes pride in my accomplishments*.*’*

#### Intention to quit (ITQ)

ITQ is a 7-point likert, single item measure that asked responders the extent to which they agree or disagree that they often think about quitting their job.

### Analyses

The SPSS 24.0 software package was used to organise and clean the dataset, as well as being used to generate the correlations, regressions that appear within the results section.

The effect of moderation and mediation was tested with Structural Equation Modelling (SEM). SEM analysis was conducting with the Lavaan package ([38]; version 0.5–20) in R (version 3.3.0) was used. SEM utilises a confirmatory approach in order to assess the structural interrelations and interactions between variables within the phenomenon, using theory to shape models that attempt to explain variance in the data. Maximum Likelihood was used for parameter estimation, as this has been deemed most appropriate for multivariate normal data and sample sizes are greater than 200 [39]. As there is no consensus within the literature as to which measure of goodness of fit is best, researchers have advised to use multiple tests [40]. The main indices that will be examined are RMSEA, where values of .08–0.05 represent adequate fit, and lower than .05 represent excellent fit [41]. Comparative fit index (CFI) was also used, where values greater than .95 are considered an excellent fit of the data [42]. Finally, the Tucker-Lewis Index was assessed, where values over .90 are considered acceptable [43].

## Results

### Correlations and regressions

Table 2 show the correlation matric between the 11 HDS variables, ITQ, and POS. POS was significantly correlated with eight of the 11 HDS traits (*Excitable*, *Sceptical*, *Cautious*, *Reserved*, *Leisurely*, *Mischievous*, *Diligent*, and *Dutiful*). *Bold* (*r* = .06; *p* = .20), *Colourful* (*r* = .04; *p* = .43) and *Imaginative* (*r* = -.07; *p* = .14) were not significantly related to POS. ITQ also was significantly correlated with eight of the 11 HDS personality traits (*Excitable*, *Sceptical*, *Cautious*, *Reserved*, *Leisurely*, *Mischievous*, *Imaginative*, and *Colourful*). However, *Bold* (*r* = -.06; *p* = .18), *Diligent* (*r* = .03; *p* = .53) and *Dutiful* (*r* = -.03; *p* = .48) were not significantly related to ITQ. A significant negative correlation was noted between POS and ITQ.

**Table 2:** Correlations between the 11 HDS traits, POS, and ITQ.

1	1	2	3	4	5	6	7	8	9	10	11	12	13
1. Excitable	1												
2. Sceptical	.28**	1											
3. Cautious	.45**	.23**	1										
4. Reserved	.29**	.28**	.26**	1									
5. Leisurely	.22**	.42**	.36**	.21**	1								
6. Bold	-.06	.28**	-.21**	-.07	.23**	1							
7. Mischievous	.03	.31**	-.15*	.06	.25**	.39**	1						
8. Colourful	-.12*	.07	-.31**	-.19**	.09	.47**	.43**	1					
9. Imaginative	.13*	.30**	.03	.11*	.28**	.37**	.40**	.29**	1				
10. Diligent	.03	.26**	.10*	-.05	.23**	.30**	.03	-.03	.16*	1			
11. Dutiful	.12*	.02	.22**	-.13*	.14*	-.02	-.12*	-.06	-.03	.19**	1		
12. POS	-.28**	-.28**	-.24**	-.27**	-.24**	.06	-.11*	.04	-.07	.10*	.12*	1	
13. ITQ	.33**	.22**	.24**	.10*	.22**	-.06	.12*	-.09*	.12*	-.03	-.03	-.46**	1

* *p* < .05;

** *p* < .001

Two hierarchical regressions were conducted to explore the relationships between the 11 HDS variables in explaining variance in POS and ITQ. These were done to provide preliminary insight into the relationships between these variables and guide the successive SEM analysis.

Table 3 shows the results of the first two-step hierarchical regression on POS. Age and gender were inserted in the first step, neither of which were significant predictors. The second step, which included the 11 HDS variables, accounted for 22% of the variance in POS. Excitable, Sceptical, Reserved, and Leisurely personality traits were found to significantly, negatively predict POS, while Diligent and Dutiful were positive predictors.

**Table 3:** Regression of age, gender, and HDS dark side as predictors of POS.

		POS	
		*β*	t
Step 1	Age	-.02	-.359
	Gender	-.09	-1.84
*F-Score*	*F(2*, *448) = 1*.*70*		
*R*^*2*^	.*008*		
Step 2	Excitable	-.15	-2.98*
	Sceptical	-.19	-3.56**
	Cautious	-.10	-1.75
	Reserved	-.10	-1.97*
	Leisurely	-.15	-2.79*
	Bold	.08	1.51
	Mischievous	-.05	-1.01
	Colourful	-.01	-.171
	Imaginative	.04	.845
	Diligent	.13	2.69*
	Dutiful	.15	3.23*
*F-Score*	*F(13*, *437) = 9*.43**		
*R*^*2*^	.*219*		
*ΔR*^*2*^	.*211*		

** = *p* < .01;

* = *p* < .05

Table 4 shows the results of the second hierarchical regression on ITQ. As with the first regression, age and gender were inserted in the first step and neither were significant predictors. The second step of the 11 HDS variables accounted for 18.5% of the variance in ITQ. Excitable, Sceptical, Leisurely, Mischievous and Colourful personality traits were found to significantly, positively predict ITQ whilst Reserved was a significant negative predictor.

**Table 4:** Regression of age, gender, HDS dark side, and POS as predictors of ITQ.

		ITQ	
		*β*	t
Step 1	Age	.07	1.56
	Gender	.04	.834
*F-Score*	*F(2*, *448) = 1*.*46*		
*R*^*2*^	.*006*		
Step 2	Excitable	.25	5.01**
	Sceptical	.11	1.99*
	Cautious	.07	1.18
	Reserved	-.10	-2.07
	Leisurely	.14	2.57
	Bold	-.09	-1.63
	Mischievous	.12	2.15*
	Colourful	-.11	-1.97*
	Imaginative	.05	1.07
	Diligent	-.08	-1.61
	Dutiful	-.09	-1.91
*F-Score*	*F(13*, *437) = 8*.69**		
*R*^*2*^	.*185*		
*ΔR*^*2*^	.*178*		

** = *p* < .01;

* = *p* < .05

### Structural model

SEM was used to analyse a model with both moderation and mediation in order to explore how POS can impact the relationship between dark side personality (represented by the 11 HDS dark side traits) and intention to quit. The model analysed whether this relationship is mediated by POS—i.e. is the impact of personality on ITQ contingent upon and filtered by the perception of organisational support—or whether this relationship instead is moderated by POS—ITQ emerges as an interaction between dark personality and perceived organisational support.

In the model, the 11 dark side personality traits were treated as observed variables because their item-level data was not available. Perceived Organizational Support on was treated as a latent variable. Due to ITQ being a single-item measure and the unavailability of item-level data of the HDS traits, the 11 dark side personality traits and intent to quit were entered in as observed variables. As only one factor emerged when an EFA was run on the POS variable, all 9-items of the POS scale were used to represent the latent variable of perceived organizational support. This variable represents an individual’s perception of how supported they feel by their organization.

Eleven moderation terms were also put into the model and represented the interaction between each of the 11 HDS traits and POS. The variables used in this interaction were mean centred before calculating an interaction term. This method has been found to be most appropriate when analysing interaction terms in regressions and SEM [40]. The interaction terms of the dark personality traits and POS were regressed onto intent to quit to look at the moderating role of POS.

Non-significant relationships were taken out of the model in a stepwise manner until only significant variables remained. The HDS traits Bold and Imaginative were removed entirely as they were not significant predictors of intent to quit or POS. Cautious*POS and Mischievous*POS proved to be the only significant moderators terms.

**Fig 1:**
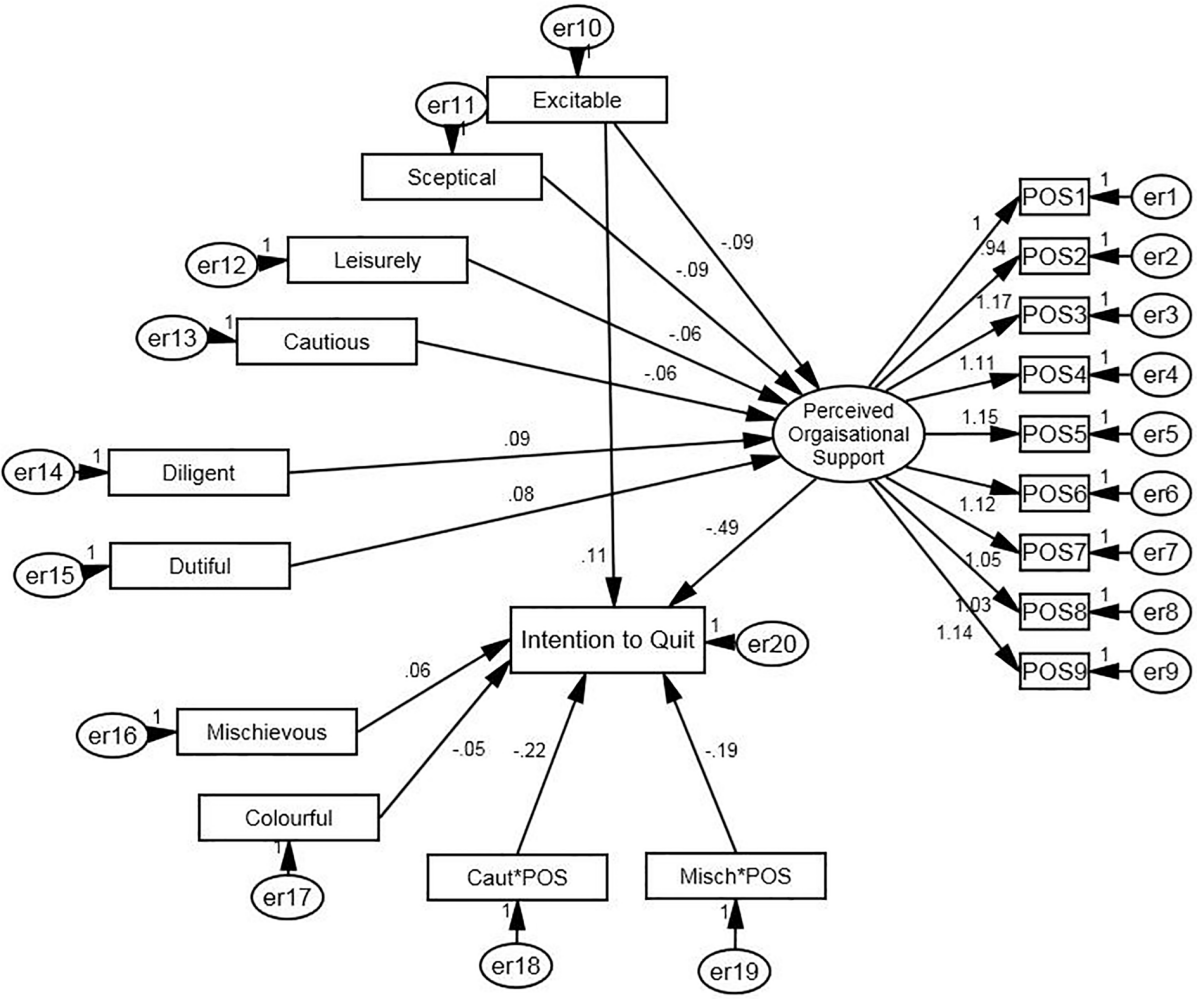
SEM testing the mediating and moderating role of POS on the relationship between dark personality and Intention to quit.

The results of this model are shown in Fig 1. A significant chi-square statistic was generated by the model (χ2(124) = 187.66, p < .001). This implies that the model differs significantly from the data structure. However, researchers have noted that chi-square values may be artificially inflated by large sample sizes, causing a rejection of the model [44]. As a result, other indices should be looked at to determine the fit of the model. These other indices suggested that the model was an excellent fit of the data: TLI = .97; CFI = .98; and RMSEA = .036 (90% CI upper limit = .025; lower limit = .046).

Excitable and Mischievous were shown to have a significant, direct positive effect on ITQ, whilst Colourful and the two moderating terms were found to have a negative impact. As anticipated, POS had a negative effect on intent to quit. The mediating role of POS was analysed by investigating the indirect effect of HDS traits on Intent to Quit. Past research has suggested that in order to assess whether the indirect effects are significant, bootstrapping procedures should be implemented [45]. As recommended by Cheung and Lau [46], 1000 bootstrap samples were created at a 95% confidence interval. Using the bias-corrected percentile method, POS was found to significantly partially mediate Excitable (β = .04; *p* = .004) on intention to quit, whilst fully mediating Cautious (β = .03; *p* = .016), Sceptical (β = .04; *p* = .002), Leisurely (β = .03; *p* = .034), Diligent (β = -.04; *p* = .005) and Dutiful (β = -.04; *p* = .002). These results suggest that POS plays a role in mediating the impact of Excitable, Cautious, Sceptical, Diligent and Dutiful dark personality traits on Intent to Quit.

## Discussion

### What does dark personality tells us about POS

This study is one of the first to look at the role of personality, particularly dark traits, in relation to POS. The results suggest that the development of POS is contingent on more than just situational factors. Individual differences that influence how employees respond to and interpret the working environment were found to explain a significant proportion (22%) of how supportive an employee perceives their organisation to be.

In particular, it was found that emotionally volatile (*Excitable*), distrusting (*Sceptical*), emotionally aloof (*Reserved*), and passive-aggressive (*Leisurely*) personalities were likely to have lower perceptions of organisational support. Employees high on Excitable are more sensitive to feeling betrayed, having short-lived enthusiasm for both people and projects. As such, they are likely to hinder their ability to develop and maintain the relationships needed for a sense of POS. Additionally, Sceptical personalities are distrusting and cynical of others, often interpreting other’s motivations as malevolent and undermining. This scepticism is likely to prevent employees from seeing their organisation as supportive, instead reinterpreting support as malicious. Reserved personalities lack interested in, and an awareness of, the feelings of others, making them socially aloof and uncommunicative. This emotional detachment appears to prevent them from experiencing the positive reciprocal affect associated with POS. Finally, Leisurely employees are passive-aggressive and tend to resist work. Leisurely personalities may therefore experience less POS as they view the reciprocal obligation (associated with POS) as a burden rather than a sign of support.

Dark personality was also found to have a positive impact on POS, with overly conscientious (*Diligent*) and dependent (*Dutiful*) personalities being more likely to perceive their organisation as supportive. Previous research has found that employees who score high on these traits tend to be easy going and have a task-orientated approach to dealing with stress. As such it appears that employees high on these *Moving Towards Others* traits experience greater POS as a function of being more likely to interpret the actions of their organisation in a positive light. This is supported by what has been suggested in previous research (i.e. [14]), as traits underpinned by positive affect are more likely to form relationships that foster POS.

### What does dark personality tell us about ITQ

This study also found that emotionally volatile (*Excitable*), distrusting (*Sceptical*), and exploitative (*Mischievous*) personalities were more likely to have intentions to leave their organisation. Excitable personalities are likely to have great ITQ as a function of their sensitivity to betrayal and short-lived enthusiasm. Additionally, previous research has demonstrated the powerful role that employee trust (or a lack of) has on ITQ, either as a function of decreased supervisor trust [47] or perceptions of organisational justice [48]. This supports the finding that Sceptical personalities, who experience greater distrust and cynicism, have higher ITQ. The Mischievous personality on the other hand is predisposed to risk taking behaviour and pushing the limits [36]. They need excitement, which might make the prospect of leaving one workplace for another one might be tempting and therefore increase the likelihood that they will have quitting intentions.

As was seen with POS, certain dark personality traits were also found to have a positive influence on turnover intentions. People with Colourful traits tend to display highly expressive, animated, and want to be noticed [49]. Previous research has demonstrated that Colourful personalities are more likely to be transformational leaders [50] and are quicker to be promoted at work [51], which could influence their willingness to stay.

### POS: A moderating and mediating effect on ITQ

The utilisation of SEM analysis offered a further detailed insight into the complex relationship between dark personality traits and ITQ. As this is the first study to examine the roles of personality and POS on ITQ simultaneously, the literature offers no foundations as to whether POS would act in a mediating or moderating capacity, so a model was generated in order to ascertain how and where POS interacts with dark personality and ITQ.

SEM analysis revealed that POS moderated the relationship between personality and ITQ. The interaction term of Cautious and POS was a significant negative predictor of ITQ. The researchers interpreted this result as personality moderating the impact of POS, with POS playing a stronger role on reducing ITQ in employees with low Cautiousness. However, further research is needed to better understand how and when this interaction occurs. Additionally, the interaction term of Mischievous and POS was found to be a significant negative predictor of ITQ. Interestingly, Mischievous personalities were previously noted to be more likely to want to leave their organisation. However, moderation analysis revealed that if POS is high, the negative impact of Mischievous is reduced.

POS was also found to be a mediator between certain dark traits and ITQ. Initial regressions indicated that the HDS trait Sceptical was a positive predictor of ITQ. However, when POS and Sceptical were assessed simultaneously in the model, Sceptical was no longer a significant predictor. Analysis of the indirect effects indicated revealed that POS fully mediated the relationship between Sceptical and ITQ. Additionally, POS was found to mediate the effects of Cautious, Leisurely, Diligent, and Dutiful on ITQ, as well as partially mediating the effect of Excitable. Previous research has demonstrated the importance of socialisation in POS and reducing ITQ [52]. This current analysis supports these results, as the *Moving Away* (characterised by withdrawal and doubting the genuineness of others) traits hinder, whilst *Moving Towards* (characterised by ingratiation) traits benefit an employee’s ability to appropriately socialise at work, influencing their willingness to perceive the organisation as supportive and caring. As *Moving Away* and *Moving Towards* reduce and increase POS respectively, this in turn influences the employee’s intention to remain at the organisation. This extends previous research, such as Woo et al.’s [30] those high in *Moving Towards* were more likely to stay with the organization. Our results indicate that *Moving Towards* traits only decreased turnover intentions indirectly, working through POS.

These findings support our hypothesis about the mediating and moderating effects of POS on turnover intention. Since the costs of employee turn are so high—both in resources and cost—identifying the antecedents of ITQ can save the organization a lot of financial and interpersonal resources (e.g. [6; 7]). This study has identified that both individual difference variables and workplace factors play a role in explaining why employees desire to leave their organisation. However, this study extends previous research by demonstrating the mediating and moderating role that POS plays. Managers that want to retain employees with traits that make them a greater risk of leaving should look to building POS as a mechanism of negating personality’s negative effect.

As with any research there are several limitations to this study. One of these is that the findings emerge from a sample that is highly androcentric (90% male) and from one industry. However, this skewed gender representation has been shown to be proportional to what is seen in the paramedic services [53], with female employees in this industry representing a larger proportion of in-hospital emergency services than ambulance-related services. That being said, future research should look to investigate the role of dark personality and POS on ITQ both in other industries and with a more evenly represented sample.

Another limitation that this study has is the scale used to measure turnover intention: a single-item measure. As a result, it might limit its reliability and effectiveness of measuring turnover intention in the participants. Therefore, it would be worth it to repeat the study with a more comprehensive measurement of turnover intention.

This was, like many others a cross-sectional study based on self-report. This means there is common-error variance that may inflate scores. Also ideally turnover is a process that should be studied longitudinally so that causal relations can be established. Thus it would be ideal to look not only at intention to quit but also when and how people do leave the organisation (resign, retire, sacking). Such studies are very rare but most desirable.
